# In response to “Antidepressants and lethal violence in the Netherlands” by D. Healy and T. Dehue

**DOI:** 10.1007/s00213-012-2767-0

**Published:** 2012-06-17

**Authors:** Paul F. Bouvy, Marieke Liem

**Affiliations:** 1 Department of Psychiatry, Erasmus Medical Centre, Postbus 2040, 3000 CA Rotterdam, The Netherlands; 2 Institute of Criminal Law & Criminology, Faculty of Law, Leiden University, Postbus 9520, 2300 RA Leiden, The Netherlands

In their letter, Healy and Dehue describe several issues that might cast doubt on the soundness and the conclusion of our study on antidepressants and lethal violence (Bouvy and Liem 2012). We are of the opinion that some of the issues brought forward are simply not about our study, some are only partially relevant and some are incorrect.

It is quite surprising to read the statement that the Dutch television program Tros Radar (broadcast in November 2009) was broadcasted “before the possible relation between antidepressant consumption and aggression was widely discussed in the Dutch media”, since Dehue wrote a large article on the subject in a leading national Dutch newspaper in June 2009 [Dehue T, van Grootheest K. Pil tegen depressie kan leiden tot moord (Drug against depression can lead to murder) NRC, 20 June 2009].

The authors’ comment on the falling numbers of young men in most Western populations, leading to lower rates of violence, may be true in general. In our study, however, men aged between 15 and 30 years were analysed as a separate group. In addition, we used ratios instead of absolute numbers.

They further suggest that autopsy rates have declined since the early 1980s, and that this decline may play a role in the decline in the total number of suicides, but we fail to see how this can have affected the number of homicides. Our study shows a striking parallel in results for both suicide and homicide.

Healy and Dehue are absolutely right in their statement that the associations shown in our study cannot be regarded as causal relations. One must be careful with interpreting these associations and the risk of ‘ecological fallacy’ can never be ruled out in a single study. However, the way in which Healy and Dehue disqualify our study by referring to the debate in the 1960s on the risks of cigarette consumption is misleading. Suggestions linking antidepressants to suicide and homicide are based on case reports and datasets on reported adverse effects. Studies based on these datasets have their own methodological problems. The issue at stake here is whether these suggestions need confirmation from other lines of research. We believe they do. One of them is epidemiology. The claim that antidepressants can cause lethal violence would gain credibility if we had found a positive association instead of a negative one. In the case of cigarette smoking, in the end, the different lines of research pointed in the same direction (Talley et al. 2004).

Our results on suicide are in line with previous research; our results on homicide are new and need replication in other populations. The future will show whether our study is an ecological fallacy or not. However, if other factors were to cause the associations we found, these other factors must have a much larger effect on suicide and homicide than the consumption of antidepressants.
